# Blockade of IL-1 family cytokines in the treatment of rheumatoid arthritis

**DOI:** 10.3389/fphar.2025.1577628

**Published:** 2025-05-30

**Authors:** Kexin Wang, Haoge Luo, Liping Liu, Hang Gao, Yanyan Song, Dong Li

**Affiliations:** ^1^ The Second Hospital of Jilin University, Jilin University, Changchun, China; ^2^ Department of Immunology, College of Basic Medical Sciences, Jilin University, Changchun, China; ^3^ Department of Bone and Joint Surgery, The First Hospital of Jilin University, Jilin University, Changchun, China; ^4^ Department of Nephrology, The Second Hospital of Jilin University, Jilin University, Changchun, China

**Keywords:** rheumatoid arthritis (RA), IL-1, autoimmune diseases, cytokines, immunotherapy, monoclonal antibody

## Abstract

Rheumatoid arthritis (RA), a chronic autoimmune disorder, imposes a substantial global health burden through elevated disability rates, systemic complications, and socioeconomic consequences. Chronic synovitis and progressive joint destruction characterize this disease, driven by dysregulated innate and adaptive immune responses that amplify synovial inflammation, osteoclastogenesis, and irreversible tissue damage. Aberrant activation of interleukin (IL) -1 family cytokines critically contributes to RA pathogenesis. These cytokines mediate dual mechanisms: pro-inflammatory agonists like IL-1β, IL-18, and IL-36 accelerate disease progression, whereas insufficient levels of anti-inflammatory antagonists such as IL-1Ra and IL-37 disrupt the balance required to suppress pathogenic cascades. Clinical trials evaluating IL-1-targeting biologics—including anakinra and canakinumab—have demonstrated robust early efficacy. However, late-stage interventions exhibit diminished therapeutic returns, largely due to irreversible joint damage and compensatory activation of redundant cytokine networks. These findings emphasize the need for precise patient stratification. Single-pathway IL-1 inhibition faces inherent limitations, driving the development of multi-target strategies to counteract cytokine redundancy and reduce therapeutic resistance. This review systematically analyzes the mechanistic roles of IL-1 family cytokines in RA, evaluates clinical outcomes and safety profiles of IL-1-targeted therapies, and proposes innovative strategies to advance RA treatment.

## 1 Introduction

Rheumatoid arthritis (RA) is a multifactorial chronic autoimmune disease primarily characterized by joint involvement in the hands, wrists, feet, ankles, knees, shoulders, and elbows. Common symptoms include chronic joint pain, stiffness, tenderness, fever, and swelling, leading to limitations in daily activities and low quality of life (Long et al., 2022). According to statistics, 18 million people worldwide were affected by RA in 2019 (Collaborators, 2020). Approximately 70% of RA patients are female, 55% are over the age of 55, and 13 million RA patients can achieve improvement through treatment (Cieza et al., 2021).

The pathogenesis of RA involves a multistep cascade of immune dysregulation, stemming from aberrant interactions between innate and adaptive immunity. This process is mediated by genetic polymorphisms, epigenetic modifications, and environmental triggers, which collectively induce synovial inflammation, hyperplasia, angiogenesis, and cartilage degradation, ultimately leading to irreversible bone destruction (Jang et al., 2022). Cytokines, particularly pro-inflammatory mediators, such as tumor necrosis factor-alpha (TNF-α), interleukin (IL) -1, IL-6, and IL-17 are central to RA progression by amplifying inflammatory responses and tissue damage (Kugler et al., 2023). Targeted therapies, including TNF-α inhibitors and IL-6 receptor antagonists, effectively reduce inflammation and improve outcomes through cytokine blockade (Smolen, 2020). However, treatment resistance persists in part of patients despite these advances. Notably, IL-1 has emerged as a critical driver of bone erosion via nucleotide- binding oligomerization domain 3 (NLRP3) inflammasome activation, underscoring its therapeutic potential for refractory RA and structural damage mitigation.

Cytokines of the IL-1 family are crucial factors in regulating immune responses (Broderick and Hoffman, 2022). This family comprises both pro-inflammatory mediators—including IL-1α, IL-1β, IL-18 and IL-33—and anti-inflammatory counterparts, such as IL-37 and IL-38 (Vincent, 2019). The intricate interplay between these cytokines and their contributions, relative to the well-established actions of TNF-α and IL-6, highlights a unique pathogenic axis that may not be fully addressed by current therapeutic strategies. The IL-1 family cytokines have mechanisms that are not covered by TNF and IL-6 (for example, IL-1β drives synovial inflammation and bone erosion by activating NLRP3 inflammasomes, while IL-37 exerts its anti-inflammatory effects by inhibiting NLRP3 activity) (Tseng et al., 2022). Research on IL-1 antagonists fills the gap left by TNF-α/IL-6 inhibitors, especially in controlling structural damage, treating refractory cases, and managing comorbidities, offering unique value. Consequently, targeting IL-1 family members offers a complementary approach that could mitigate the inflammatory cascade in RA more comprehensively.

This review provides a detailed overview of recent investigations into the roles of IL-1 family cytokines in RA pathogenesis and critically examines the current status of drugs targeting these cytokines in clinical trials, thereby offering novel perspectives on the treatment of rheumatoid diseases.

## 2 Etiological factors of RA

### 2.1 Genetic factors

Genetic factors are important risk factors. Over 100 genetic loci have been identified to influence the development of RA through regulating immunity and other pathways (Padyukov, 2022). The genetic predisposition to RA is strongly linked to *HLA-DRB1* alleles, particularly in anti-citrullinated protein antibody (ACPA)-positive subtypes. HLA-DRB1*04 and HLA-DRB1*10, the primary risk alleles for ACPA-positive RA, encode valine (Val) at position 11, forming the “shared epitope” (SE) (Raychaudhuri et al., 2012). These alleles confer a markedly elevated risk (odds ratio, OR = 3.88–10) by binding citrullinated peptides to activate T cells and initiate autoimmune cascades (Irigoyen et al., 2005; van der Woude et al., 2010). Smoking interacts synergistically with HLA genes, amplifying genetic risk in SE allele carriers (Stolt et al., 2003). Conversely, HLA-DRB1*13 (encoding glutamic acid [Glu] at position 71) demonstrates a protective effect against ACPA-positive RA (Terao et al., 2019). In contrast, ACPA-negative RA exhibits weaker genetic associations, primarily linked to the presence of leucine (Leu) or serine (Ser) at position 11 of HLA-DRB1 (Bossini-Castillo et al., 2015). Secondary HLA loci also contribute: *HLA-B* (Asp at position 9; OR = 2.12), *HLA-DPB1* (Phe at position 9; OR = 1.40), and HLA-A (Asn at position 77; OR = 0.85) show modest effects, with *HLA-A* suggesting a protective trend (Regueiro et al., 2021).

What’s more, the genetic risk of RA shows heterogeneity across different populations. *HLA-DRB1* alleles exhibit distinct risk profiles across ethnic groups. European ACPA-positive RA predominantly associates with *HLA-DRB1*04 and *HLA-DRB1*10 (OR = 3.88), whereas Japanese populations show *HLA-DRB1*09 (Asp at position 11) as the primary risk allele, with independent contributions from *HLA-DQ* loci (Raychaudhuri et al., 2012; Okada et al., 2016). In Han Chinese, *HLA-DQA1* (Asp at position 160) independently correlates with ACPA-positive RA, underscoring *HLA-II* molecules’ role in antigen presentation (Guo et al., 2019). The *HLA-DQB1*03:02 allele reduces ACPA-positive RA risk in Malaysians (OR = 0.85), likely via T-cell receptor signaling modulation (Tan et al., 2021). Among African Americans, *HLA-DRB1*04 demonstrates strong RA risk association (OR = 2.12), though its population frequency and epistatic interactions require validation (Danila et al., 2017). These findings collectively highlight HLA-driven RA pathogenesis through antigen presentation and immune dysregulation. Future cross-population multi-omics studies should clarify genetic heterogeneity’s implications for precision medicine.

The genetic architecture of RA extends beyond HLA genes to encompass over 150 non-HLA loci that orchestrate immune regulation, inflammatory signaling, and cellular activation pathways (Padyukov, 2022). Rong et al. demonstrated that specific *IL1B* polymorphisms differentially modulate RA risk in Han Chinese populations: the rs1143643 variant confers protection, whereas rs16944 elevates susceptibility (Rong et al., 2020). Zhu et al. further identified significant associations between *IL-1A* +4845G/T polymorphisms and RA susceptibility across populations (dominant model: p = 0.02; recessive model: p = 0.05; allelic model: p = 0.04), while *IL-1B* +3954C/T polymorphisms showed population-specific effects, demonstrating global significance in general populations (recessive model: p = 0.03; allelic model: p = 0.01) and enhanced association in Asian cohorts (recessive model: p = 0.007; allelic model: p = 0.002) (Braga et al., 2024). Beyond these loci, multiple genes critically contribute to RA pathogenesis. The *PTPN22* rs2476601 variant (OR = 1.81, as reflected in pooled meta-analyses) in European populations augments autoreactive T-cell activation through impaired T-cell receptor signaling via lymphocyte tyrosine phosphatase (Begovich et al., 2004). *CTLA4* mitigates autoimmune risk (OR = 0.88) by suppressing T-cell co-stimulation, particularly in ACPA-positive subtypes (Stahl et al., 2010). *STAT4* rs7574865 potentiates pro-inflammatory cytokine signaling (IL-12/IL-23) with risk alleles (OR = 1.16) showing trans-ethnic associations (Okada et al., 2012). *TNFAIP3* rs10499194 (OR = 1.33) drives inflammation through dysregulated NF-κB inhibition, strongly correlating with ACPA-positive RA (Eyre et al., 2012). *PADI4* rs2240336 facilitates protein citrullination and subsequent ACPA production (OR = 0.88), exhibiting heightened significance in Asian populations (Eyre et al., 2012). The *IL6R* rs2228145 protective variant (OR = 0.93) modulates inflammatory cascades through IL-6 receptor regulation (Okada et al., 2014). Additional contributors include *TRAF1-C5* (inflammatory signaling), *CCR6* (T-cell chemotaxis), and *IRF5* (interferon response), collectively underscoring the polygenic nature of RA susceptibility (Stahl et al., 2010).

### 2.2 Environmental factors

The pathogenesis of RA is closely related to genetic factors and involves the participation of various environmental factors. Among these environmental factors, smoking is central, significantly interacting with genetic susceptibility (especially HLA genes) (Wouters et al., 2022). Other factors, such as infections, hormones, and occupational exposures, contribute to disease onset through different mechanisms.

Tobacco smoking remains the most well-characterized environmental risk factor for RA, demonstrating particular association with APCA-positive subtypes. Cigarette smoking synergistically interacts with HLA-DRB1 SE alleles (*04, *10) to promote RA risk, with smokers carrying a single SE allele exhibiting an OR of approximately 3.8 (Padyukov et al., 2004). Tobacco-derived chemicals including nicotine and tar induce protein citrullination through enzymatic modification, thereby initiating ACPA production and subsequent autoimmune cascades. These compounds directly stimulate immune cell activation, enhancing TNF-α and IL-17 secretion while impairing Treg cell functionality, ultimately disrupting immune homeostasis. Occupational hazards, notably chronic silica dust and formaldehyde exposure, exert pro-inflammatory effects through alveolar macrophage activation (Klareskog et al., 2020). This process triggers IL-1β and TNF-α release, propagating systemic inflammatory responses that elevate RA susceptibility. Environmental pollutants such as PM2.5 and NOx compounds demonstrate dose-dependent RA risk enhancement, particularly in genetically predisposed populations (Zhang et al., 2023). These particulates primarily activate the NF-κB signaling pathway, stimulating synovial cells to secrete inflammatory mediators including IL-1β and TNF-α, which accelerate joint inflammation and potentiate osteodestructive processes.

Dietary modifications alter gut microbiota composition and dynamics, inducing dysbiosis that sustains systemic pro-inflammatory states (Lin et al., 2023). Vitamin D deficiency impairs immune tolerance through disrupted Treg cell regulation, elevating autoimmune disease susceptibility (Charoenngam, 2021). Elevated sodium chloride intake drives the polarization of pathogenic TH17 cells, particularly under conditions mimicking interstitial sodium concentrations, which amplifies IL-17A and GM-CSF production to exacerbate inflammatory cascades. Adipose tissue dysfunction elevates reactive oxygen species (ROS) generation while stimulating adipocyte-derived pro-inflammatory cytokines including TNF-α and IL-6, thereby accelerating systemic inflammation and RA progression (Braga et al., 2024).

Pathogen-induced RA manifests through gene-environment interactions, where PTPN22 or STAT4 genetic variants lower infection response thresholds to specific microbes (Jang et al., 2022). Periodontal pathogens like *Porphyromonas gingivalis* induce protein citrullination via peptidylarginine deiminase enzymes, generating autoantigens that cross-react with ACPA (Kobayashi and Bartold, 2023). While Epstein-Barr virus (EBV) exhibits epidemiological associations with RA, mechanistic evidence remains limited; proposed pathways include EBV nuclear antigen-1 molecular mimicry of citrullinated fibrinogen, which may activate autoreactive T-cell clones and synovial antibody production (Costenbader and Karlson, 2006).

### 2.3 Immune factors

The aberrant activation of the immune system plays a central role in the pathological progression of RA. Immune dysregulation is predominantly characterized by an imbalance in CD4^+^/CD8^+^ T-cell subset ratios and disruption of the cytokine network. In most patients, CD4^+^ T cells predominate over CD8^+^ T cells and secrete cytokines that influence the differentiation and activation of other immune cells, including CD8^+^ T and B cells. While CD8^+^ T cells contribute to the inflammatory milieu, their role is generally considered secondary to that of CD4^+^ T cells in driving immune dysregulation in RA (Jang et al., 2022). Within the synovial membrane in RA, CD4^+^ T cells serve as the primary drivers of inflammatory responses and disease relapse, while CD8^+^ T cells play a secondary role in disease pathogenesis. CD4^+^ T cells can produce IL-17, activate Th17 cells and inhibit Treg cells. CD8^+^ T cells secrete IFN-γ, CCL5 and other cytokines that enhance the inflammatory microenvironment (Jonsson et al., 2022). These cytokines act on CD14 mononuclear precursor cells of osteoclasts, influencing the differentiation or maturation of osteoclasts via the receptor activator of nuclear factor κB (RANK)/ receptor activator of nuclear factor κB ligand (RANKL)/osteoprotegerin (OPG) signaling pathway (Aletaha and Smolen, 2018). Under inflammatory conditions, myeloid lineage cells such as macrophages release substantial TNF-α upon stimulation by IL-17 and IFN-γ, a pivotal cytokine driving bone erosion through direct activation of osteoclast differentiation and concurrent suppression of osteoblast functionality (Wang and He, 2020). Clinically employed anti-TNF-α biologics like infliximab demonstrate efficacy in mitigating osteoclast-mediated joint destruction and attenuating inflammatory responses in RA. However, persistent impairment of osteoblast-driven bone formation remains unresolved (Malviya et al., 2009; Kwon et al., 2017). This therapeutic limitation underscores the necessity to explore complementary pathways. Notably, IL-1 family cytokines (IL-1α/IL-1β) exhibit potent osteoclastogenic effects via the RANK/RANKL/OPG signaling axis, operating independently of TNF-α-mediated pathways (Son et al., 2020). Mechanistically, IL-1 enhances RANKL expression in osteoblasts and stromal cells, amplifying osteoclast precursor differentiation through NF-κB and MAPK activation. IL-1Ra inhibits osteoclastogenesis (Izawa et al., 2014; Zou et al., 2021). While TNF-α inhibitors primarily address inflammatory osteolysis, IL-1 blockade concurrently enhances osteoanabolic processes and suppresses bone resorption. This multi-target strategy could overcome the limitations of monotherapy by simultaneously protecting against structural joint damage and facilitating bone repair in RA.

B cells play a pivotal role in the pathogenesis and progression of RA. Antigen presenting cells (APCs) can recognize and present immune complexes, activating CD4^+^ T helper cells and B cells via co-stimulation. Activated B cells secrete both inflammatory and regulatory cytokines, form ectopic germinal centers and activate T cells via antigen presentation and co-stimulatory molecules (Wu et al., 2021). In parallel, B cells produce various antibodies, including rheumatoid factor (RF) antibodies targeting the Fc portion of immunoglobulin G (IgG), and anti-citrullinated protein antibodies (ACPA) targeting citrullinated proteins (Xiao et al., 2021). Immune complexes formed through antigen-antibody binding accumulate in the synovial fluid, activating osteoclasts to cause bone damage. They also release cytokines such as tumor TNF-α and IL-8, inducing immune cell infiltration and exacerbating chronic inflammation in the joints (Maeda et al., 2022). The IL-1 family modulates B-cell activity and antibody production (Boraschi et al., 2018). Pro-inflammatory cytokines within the IL-1 family, such as IL-1 and IL-18, directly stimulate B-cell activation and promote the production of antibodies like RF and ACPA (Mateen et al., 2016). Current Phase III clinical trial results indicate that IL-1 blockade therapy significantly reduces ACPA and RF levels in patients’ serum and is positively correlated with the normalization of B-cell subset ratios, suggesting that targeting the IL-1 family may be a strategy for regulating abnormal B-cell activation and improving the course of RA (Winkler et al., 2021).

RA extends beyond localized joint pathology to manifest as a systemic autoimmune disorder. RA patients exhibit immune system dysregulation characterized by self-reactive tissue destruction. While hallmark rheumatic features including joint swelling, pain, and stiffness dominate clinical presentation, multi-organ involvement frequently develops. Cutaneous manifestations range from subcutaneous rheumatoid nodules to vasculitic lesions; pulmonary complications include progressive interstitial lung disease; cardiovascular risks escalate through accelerated atherosclerosis. Secondary Sjögren’s syndrome, characterized by xerophthalmia and xerostomia, develops in approximately 19.5% of RA patients (Alani et al., 2018). Additionally, hematologic disturbances (e.g., anemia of chronic disease) and peripheral neuropathies frequently accompany advanced disease (Conforti et al., 2021). This multi-system pathophysiology necessitates comprehensive management strategies targeting both synovial inflammation and extra-articular sequelae. Modern therapeutic paradigms combine immunomodulatory biologics/JAK inhibitors with proactive monitoring for cardiopulmonary and ocular complications to optimize long-term outcomes.

## 3 The role of IL-1 family cytokines in RA

The IL-1 family of cytokines may have overlapping and complementary effects in promoting or inhibiting the development of RA. Within the IL-1 family, IL-1α, IL-1β, IL-18, IL-33, IL-36α, IL-36β, and IL-36γ can promote the occurrence and development of RA, while IL-1Ra, IL-36Ra, IL-37, and IL-38 can suppress inflammatory responses. The specific mechanisms of each cytokine in the IL-1 family in RA are as follows:

### 3.1 IL-1

The IL-1 cytokine family features three principal subtypes: IL-1α, IL-1β, and the endogenous antagonist IL-1Ra. Membrane-bound IL-1α drives early localized inflammation through paracrine activation of RANKL expression in adjacent stromal cells, inducing osteoclastogenesis via RANK/RANKL/OPG-independent mechanisms (Kim et al., 2009). In contrast, caspase-1-activated IL-1β exerts systemic effects by disrupting RANKL/OPG equilibrium through dual pathways - directly upregulating osteoclast RANKL production while suppressing osteoblast OPG secretion. IL-1Ra competitively blocks IL-1 receptor binding, effectively inhibiting NF-κB and MAPK signaling cascades. Chronic TLR-mediated NF-κB activation in RA synovium induces NLRP3 and pro-IL-1β co-expression. Potassium efflux, ROS overproduction, or lysosomal damage triggers NLRP3 inflammasome assembly, enabling caspase-1-mediated proteolytic cleavage and extracellular release of mature IL-1β. This bioactive cytokine binds IL-1R to amplify NF-κB/MAPK signaling in osteoclast precursors, driving NFATc1-dependent differentiation and bone resorption (Kong et al., 1999; Roux and Orcel, 2000). IL-1β simultaneously reprograms Tregs into osteoclastogenic O-Tregs through RANKL induction, effectively converting immunosuppressive cells into bone-destructive effectors (Nasi et al., 2017; Levescot et al., 2021). IL-1α and IL-1β can stimulate the proliferation of synovial fibroblasts and the release of matrix metalloproteinases (MMPs), leading to the breakdown of collagen in the cartilage matrix (Reboul et al., 1996).

IL-1β triggers inflammatory responses in fibroblast-like synoviocytes, including MH7A cells, while upregulating GATA4 expression. GATA4 directly binds to gene promoters to enhance transcriptional activation of angiogenic factors VEGFA and VEGFC (Jia et al., 2018). Through activation of the VEGFR2/PI3K/AKT pathway, VEGF stimulates synovial fibroblast proliferation and invasion, exemplified by MH7A cells, ultimately driving pathological synovial tissue thickening. Newly formed vasculature combines with hyperplastic synovium to generate an erosive pannus that progressively degrades articular cartilage and subchondral bone, culminating in irreversible joint damage (Chen et al., 2024). Furthermore, IL-1α and IL-1β can also induce articular chondrocytes to produce nitric oxide (NO) and prostaglandin E2 (PGE2), inhibiting the synthesis of matrix proteins and contributing to cartilage destruction (Pelletier et al., 1996). Additionally, IL-1β, through its ability to activate signaling pathways such as MAPK upon binding to cellular receptors, can trigger the release of other cytokines and drive the polarization of Th17 cells. This, in turn, promotes the release of IL-17 from CD4^+^ T cells, ultimately enhancing the inflammatory response (Iwahashi et al., 2004).

### 3.2 IL-18

IL-18, a pro-inflammatory cytokine of the IL-1 superfamily, predominantly exists as an inactive precursor (pro-IL-18) in macrophages, dendritic cells, and epithelial lineages. Canonical activation requires caspase-1-mediated proteolytic cleavage within the NLRP3 inflammasome, enabling secretion of mature IL-18 via gasdermin D (GSDMD) pores. During Gram-negative bacterial infections, non-immune cells bypass this pathway by directly activating caspase-4/5 to process pro-IL-18, expanding its functional repertoire in innate immunity (Landy et al., 2024).

Upon binding to its heterodimeric receptor (IL-18Rα/β), IL-18 triggers MyD88-dependent signaling cascades that activate NF-κB and MAPK pathways. These pathways drive transcriptional upregulation of IL-6, IL-8, and CXCL10 while promoting Th17 differentiation and IL-17 production (Rex et al., 2020). Synergy with IL-12 amplifies IFN-γ secretion in Th1 and NK cells, establishing a feedforward loop that sustains synovial inflammation through TNF-α, IL-1β, and MMP-mediated cartilage degradation (Baggio et al., 2023).

In RA, IL-18 exacerbates bone erosion through dual mechanisms. First, it directly modulates osteoclastogenesis by skewing the RANKL/OPG balance toward bone resorption (Zhang et al., 2013). Second, IL-18 synergizes with TNF-α and IL-6 to activate fibroblast-like synoviocytes (FLS), which secrete matrix metalloproteinases (MMPs) that degrade cartilage and subchondral bone (Puren et al., 1998; Morel et al., 2001). T cells in RA synovium further amplify osteoclast formation by increasing RANKL production under IL-18 stimulation. Preliminary evidence indicates that IL-18 bypasses RANKL dependence in certain contexts, directly inducing osteoclast differentiation and bone destruction (Kim et al., 2015). The cytokine also recruits inflammatory cells to joints via CCL2 upregulation, creating a self-perpetuating cycle of inflammation. Concurrently, IL-18 enhances Th17-mediated IL-17 and IL-22 release, accelerating synovitis and osteoclast activation (Vasilev et al., 2021).

### 3.3 IL-33

IL-33 has dual pro-inflammatory and anti-inflammatory regulatory roles in RA (Chen et al., 2019). In the early acute injury phase, it exerts protective effects by activating Tregs and type II immune responses, while in the chronic phase, pro-inflammatory signals dominate and exacerbate pathological damage.

During the acute phase, IL-33 primarily exhibits anti-inflammatory effects. Early low-dose IL-33 intervention can inhibit the progression of collagen-induced arthritis models. IL-33 enhances the proliferation and immune suppressive function of Treg cells via the STAT6 pathway, inhibits Th17 differentiation and osteoclast generation, reduces the RANKL/OPG ratio, and participates in the restoration of immune tolerance mediated by Breg cells (Biton et al., 2016). IL-33 can also induce ILC2 cells to secrete IL-4/IL-13, promoting M2 macrophage polarization to inhibit NF-κB activity (Lott et al., 2015).

In the chronic phase, IL-33 binds dimers of ST2 receptor and Interleukin 1 receptor-associated protein (IL-1RacP), which recruits IL-1RacP, MyD88, Interleukin 1 receptor associated kinase (IRAK) 1, IRAK4, and TNF receptor associated factor (TRAF) 6. This complex activates NF-κB and MAPK signaling pathways, leading to the phosphorylation and activation of extracellular signal-regulated kinase (ERK) 1/2, c-Jun N-terminal kinase (JNK), p38, and phosphatidylinositol 3-kinase (PI3K)/protein kinase B (PKB/AKT) signaling modules. Consequently, the expression of pro-inflammatory cytokines is enhanced (Cayrol and Girard, 2009; Yuan et al., 2011). In Th2 cells, IL-33 promotes the transcription of IL-4, IL-5, and IL-13 (Schmitz et al., 2005; Verri et al., 2010). IL-33 upregulates the expression of CXC motif chemokine ligand 1(CXCL1), C-C Motif Chemokine Ligand 3(CCL3), TNF-α, and IL-1β in macrophages, as well as IL-6, IL-13, IL-1β, GM-CSF, Monocyte chemoattractant protein-1 (MCP-1), and Macrophage inflammatory protein-1alpha (MIP-1α) in mast cells (Xu et al., 2008; Yuan et al., 2011). It also increases the expression of IL-6, IL-8, MCP-1, MMP-1, and MMP-3 in synovial fibroblasts, thereby promoting synovial inflammation and cartilage destruction (Kunisch et al., 2012).

Therefore, targeting the IL-33/ST2 axis in therapy requires a precise balance of its dual functions, such as developing sST2 antagonists to block pro-inflammatory signals while retaining the activation of membrane-bound ST2 on Tregs, providing a new direction for personalized treatment of RA.

### 3.4 IL-36

IL-36 has multiple subtypes, including IL-36α, IL-36β, and IL-36γ, which bind to the receptor comprising specific chain IL-36R (IL-1Rrp2) and co-receptor IL-1R accessory protein (IL-1RAcP). IL-36 can activate a cascade of downstream effectors, including IκB, NF-κB, PI3K/AKT, and ERK, leading to the regulation of inflammatory cytokine, chemokine, and growth factor production through transcription factors mTORC and Wnt5a in the cell nucleus (Zhou et al., 2018). This promotes neutrophil infiltration, dendritic cell activation, and the polarization of Th1 cells and IL-17-producing T cells (αβ T cells and γδ T cells), ultimately exacerbating the development of RA inflammation (Vigne et al., 2012; Gabay and Towne, 2015). Furthermore, IL-36 stimulates fibroblasts to produce pro-inflammatory factors (Boutet et al., 2016), and targets autophagy to regulate the proliferation, migration, and invasion of RA synovial cells (Hao and Liu, 2021). In contrast, IL-36RA antagonizes IL-36α, IL-36β, and IL-36γ by binding to IL-1Rrp2 (Towne et al., 2011; Vigne et al., 2011). IL-36RA blocks IL-1RAcP recruitment and reduces the downstream activation of NF-κB or MAPK pathways (Towne et al., 2004).

### 3.5 IL-37

The binding of IL-37 to its receptors, IL-1R8 and IL-18Rα, triggers a cascade of signaling pathways, including PI3K/AKT, ERK, JNK, and p38. This, in turn, modulates the activation of Notch1 and Nuclear factor erythroid 2-related factor 2 (NRF2) through signal transducer and activator of transcription 3 (STAT3) and single immunoglobulin interleukin-1-related receptor (SIGIRR), thereby conferring anti-inflammatory properties (Zhou et al., 2020). Furthermore, IL-37 has been shown to attenuate TNF-α-induced apoptosis of synovial fibroblasts by suppressing the NF-κB/Gasdermin-D (GSDMD) signaling axis (Ren et al., 2023).

The anti-inflammatory effects of IL-37 are attributed to its ability to modulate immune cell function. IL-37 can suppress the expression of inducible nitric oxide synthase (iNOS), IL-6, MCP-1 and reactive oxygen species (ROS) in macrophages, improve the expression of IL-10, glutathione peroxidase 4 (GPX4), NRF2, and CD206 (Qi et al., 2019; Zhao et al., 2022). Additionally, IL-37 can impede dendritic cell maturation by down-regulating the expression of MHC class II (MHCII), CD40, CD86, and CD80 (Liu et al., 2019). Notably, IL-37 can also inhibit IgG production in B cells, eosinophil infiltration, Th17 and Tfh cell differentiation and proliferation, while fostering Th1 and Treg cell differentiation (Liu et al., 2020; Li et al., 2021).

### 3.6 IL-38

IL-38 shares receptors with IL-36 and antagonizing IL-36. Binding to IL-36R or IL-1RAPL1 receptors, IL-38 triggers a signaling cascade involving Toll and interleukin-1 receptor (TIR), RohA, ERK, JNK, and p38, which in turn activates transcription factors AP-1 and the silent information regulator sirtuin 1 (SIRT1), ultimately resulting in anti-inflammatory effects in RA (Boutet et al., 2017). Notably, low levels of IL-38 can form an IL-38/36R axis, either by binding to IL-36R and blocking IL-1RAcP recruitment or by recruiting inhibitory receptors to prevent MyD88 recruitment, thereby inhibiting NF-κB or MAPK and eliciting anti-inflammatory effects (Mora et al., 2016). The IL-38/IL-1RAPL1 axis, comprising IL-38 and IL-1RAPL1, can exert anti-inflammatory or pro-inflammatory effects depending on the length of IL-38 acting on JNK/AP-1 (Mora et al., 2016; Xie et al., 2019). Additionally, IL-33 can modulate the SIRT1/HIF-1α signaling pathway to suppress inflammatory responses (Pei et al., 2020).

## 4 The receptor of IL-1 family

**FIGURE 1:**
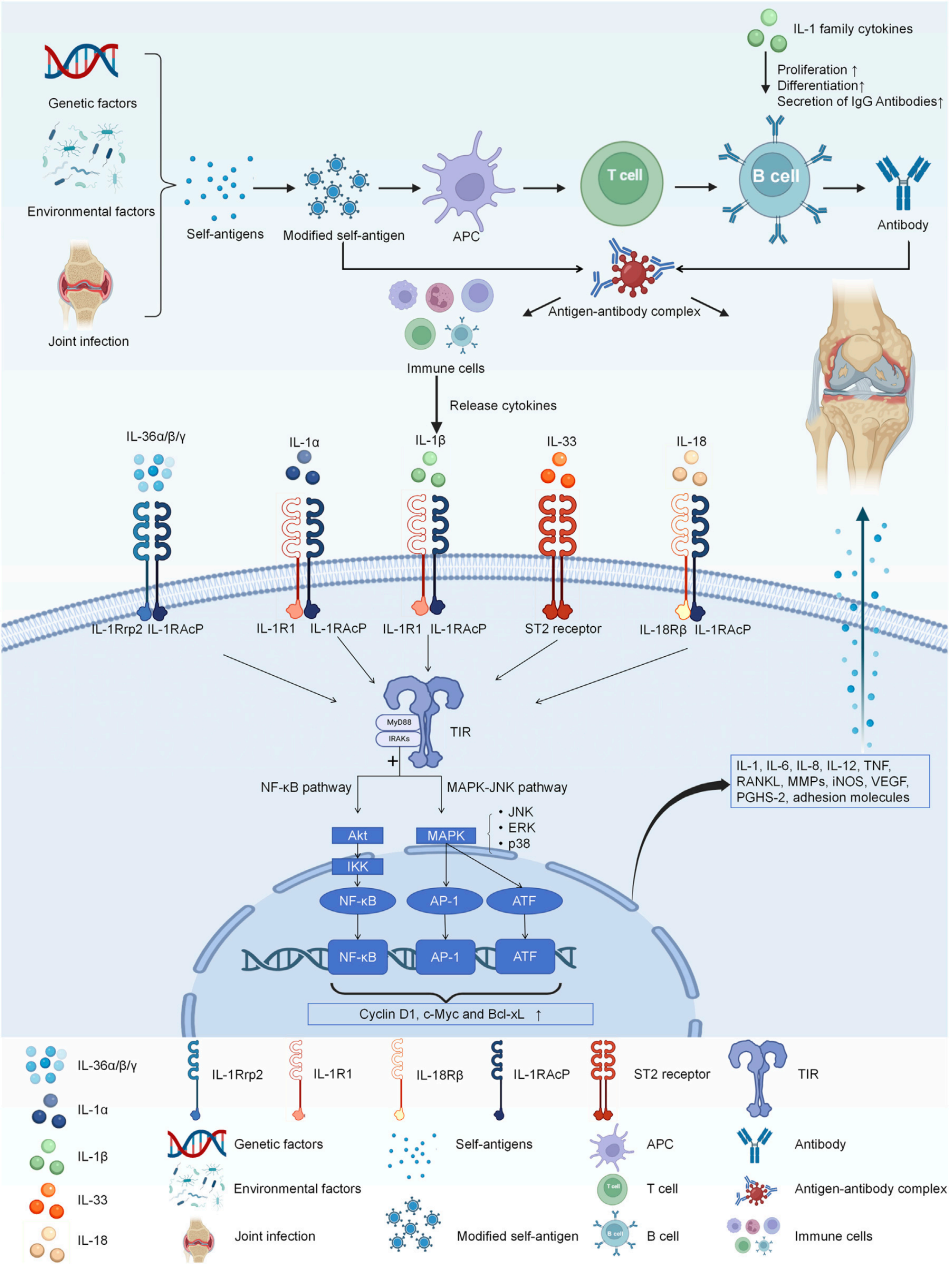
The IL-1 receptor family comprises IL-1R1, IL-1R2, IL-1RAcP, ST2, IL-18Rα, IL-1Rrp2, IL-18Rβ. Different cytokines bind to distinct receptors, and they all activate the TIR pathway. And then, MyD88 recruits IRAK to TIRs through the interaction of their death domains. IRAK is activated by phosphorylation and then associates with TRAF6, leading to the activation of two distinct signaling pathways, and finally to the activation of JNK and NF-κB. Finally they eventually have the influence on the expression of IL-1, IL-6, IL-8, IL-12, TNF, RANKL, MMP, iNOS, VEGF, PGHS-2, and adhesion molecules, exacerbating joint inflammation.

The IL-1 receptor family comprises IL-1R1 (IL-1RI), IL-1R2 (IL-1RII), IL-1R3 (IL-1RAcP), IL-1R4 (the suppression of tumorigenicity 2 receptor, ST2), IL-1R5 (IL-18Rα), IL-1R6 (IL-1Rrp2, IL-36R), IL-1R7 (IL-18Rβ), IL-1R8 (TIR8, also known as SIGIRR), IL-1R9 (TIGIRR-2), and IL-1R10 (TIGIRR-1) (Kim and Lee, 2024). Although different cytokines bind to distinct receptors, these receptors can activate the TIR pathway upon stimulation (Dinarello, 2018). Following TIR stimulation, MyD88 recruits IRAK to TIRs through the interaction of their death domains. IRAK is activated by phosphorylation and then associates with TRAF6, leading to the activation of two distinct signaling pathways, and finally to the activation of JNK and NF-κB (Zhou et al., 2022). This cascade of events culminates in the upregulation of IL-1, IL-6, IL-8, IL-12, TNF, RANKL, MMP, iNOS, VEGF, PGHS-2, and adhesion molecules, exacerbating joint inflammation (Narayanan and Park, 2015). Details are shown in Figure 1 and Table 1.

**TABLE 1:** Classification and characteristics of IL-1 family cytokines.

Subfamily	Cytokine	Function	Specific receptor	Coreceptor	Signaling pathway	Cellular source	Regulatory mechanism
IL-1 Subfamily	IL-1α	Pro-inflammatory	IL-1R1	IL-1RAcP (IL-1R3)	MyD88/NF-κB	Macrophages, epithelial cells (alarmin release)	Damage-induced release (alarmin)
	IL-1β	Pro-inflammatory	IL-1R1/IL-1R2	IL-1RAcP (IL-1R3)	NLRP3 inflammasome → Caspase-1 activation	Monocytes, dendritic cells (LPS/NLRP3 activation)	PAMP-triggered precursor cleavage
	IL-1Ra	Anti-inflammatory	IL-1R1	—	Blocking IL-1R1 dimerization	Lymphocytes, stromal cells (feedback inhibition)	IL-4/IL-13 upregulation
	IL-33	Dual-phase regulation	ST2 (IL-1R4)	IL-1RAcP (IL-1R3)	ST2-MyD88 → Th2 polarization	Fibroblasts, endothelial cells (tissue damage)	Necrotic cell release (DAMP signaling)
IL-36 Subfamily	IL-36α/β/γ	Pro-inflammatory	IL-36R (IL-1R6)	IL-1RAcP (IL-1R3)	mTORC1/Wnt5a pathway	Keratinocytes, monocytes (TLR-mediated activation)	Keratinocyte TLR activation
	IL-36Ra	Anti-inflammatory	IL-36R (IL-1R6)	IL-1RAcP (IL-1R3)	Inhibiting IL-36R dimerization	Keratinocytes (psoriasis suppression)	TGF-β-dependent expression
	IL-38	Anti-inflammatory	IL-36R/IL-1RAPL1 (IL-1R6)	IL-1R9	AP-1/SIRT1 pathway inhibition	Monocytes, Tregs, keratinocytes, adipocytes, synoviocytes	Antagonizing IL-36R signaling
IL-18 Subfamily	IL-18	Pro-inflammatory	IL-18Rα (IL-1R5)	IL-18Rβ (IL-1R7)	ASC inflammasome → IFN-γ synergy	Kupffer cells, macrophages (pathogen-induced)	Caspase-1-dependent maturation
	IL-37	Anti-inflammatory	IL-18Rα (IL-1R5)	IL-1R8	STAT3/Nrf2 activation	Tregs, epithelial cells (STAT3/Nrf2 pathway)	Epigenetic modification (HDAC regulation)

## 5 Clinical pre-study of monoclonal antibody targeting IL-1 family cytokines for the treatment of RA

There are numerous therapeutic interventions that target the IL-1 family, with a significant number having advanced into clinical research stages. The details are shown in Tables 2, 3.

**TABLE 2:** Clinical trials of biological agents targeting IL-1 family cytokines.

Drug	Mechanism	Efficacy	Adverse events	Phase	Source	Citation
PF-06650833	Inhibits IRAK4	Efficacy indicators: reduction in expression of interferon gene markers in whole blood. Inflammatory response: inhibition of inflammatory response induced by plasma from RA and SLE patients.	No serious adverse events reported; well-tolerated with no dose-limiting toxicity.	Phase I	NCT02485769	Winkler et al. (2021)
		Efficacy indicators: continuous reduction of high-sensitivity CRP in serum (≥250 mg dose). Inflammatory response: inhibition of expression of inflammatory factors.	Headache (frequent), gastrointestinal discomfort, and acneiform rash were reported. No severe events or fatalities occurred.	Phase I		Danto et al. (2019)
Anakinra	Glycosylated recombinant IL-1Ra binds to IL-1R1 and competitively inhibits the binding of both IL-1α and IL-1β to IL-1R1.	Efficacy indicators: HbA1c% significantly decreased in the anakinra group. Inflammatory response: DAS28 score improved, and the use of corticosteroids decreased.	Minor side effects (e.g., infections, injection site reactions) were observed, with no serious adverse events.	Phase IV	NCT02224651	Ruscitti et al. (2019)
		Efficacy indicators: HbA1c% significantly decreased in the anakinra group. Inflammatory response: DAS28 score decreased, CRP decreased. Metabolic improvement: The anakinra group reduced the use of antidiabetic drugs.	Only mild adverse events (e.g., injection site reactions) were documented, and no severe adverse events occurred.	Phase IV	NCT02485769	Ruscitti et al. (2021)
		Efficacy indicators: no significant difference in Larsen scores between anakinra and placebo (2.50 vs. 4.16), lower improvement in DAS28/HAQ. Inflammatory response: lower improvement in DAS28.	A higher incidence of severe adverse events was noted in the treatment group (11 vs. 6). Severe infections were reported.	Phase II		Scott et al. (2016)
		Efficacy indicators: higher remission rate (57% in anakinra group at 8 weeks vs. 50% in DMARD group). Joint erosion: DKK-1 is positively correlated with the Sharp score (bone erosion). Mechanism: TNF-α inhibitors and IL-1Ra significantly reduce DKK-1 levels, inhibiting the dysregulation of the Wnt pathway. Inflammatory response: significant improvement in SF-36 scores.	Not applicable		NCT02236481	Wang et al. (2011)
		Efficacy indicators: 43% ACR response rate; Inflammatory response: CRP, ESR significantly decreased;	Injection site reactions were prominent. No Serious Adverse Events.	Phase II		Nordström et al. (2012)
		Efficacy indicators: ACR20 46% (1.0 mg/kg group) vs. 19% (placebo); Joint erosion: significant slowing of Larsen score and erosion joint count;	Injection site reactions were prominent, with discontinuation rates of 5%. No Serious Adverse Events.	Phase II	NCT02236481	Bresnihan et al. (1998)
		Efficacy indicators: ACR20 46% (48 weeks); ACR50 18%, ACR70 3%; Inflammatory response: ESR improvement;	Injection site reactions (7%–10%); Slightly higher rate of severe infection (no opportunistic infection or death).	Phase II	ISRCTN15819795	Cohen et al. (2002)
		Efficacy indicators: ACR20 46%; ACR50 18%, ACR70 3% (48 weeks). Joint erosion: long-term delay in radiographic progression;	Injection site reaction; No new safety concerns reported with long-term use.	Phase II	ChiCTR-CCC-10001054	Nuki et al. (2002)
		Efficacy indicators: HAQ-DI significantly improved (rapid onset in high-dose group)	Injection site reactions; No significant increase in infection rate.	Phase II	NCT01033656	Cohen et al. (2003)
		Efficacy indicators: improvement of symptoms and radiographic progression	Severe infection (2.1% vs. 0.4%); Overall well-tolerated; Injection site reactions are common.	Phase III		Fleischmann et al. (2003)
		Efficacy indicators: ACR20 38% vs. 22% (placebo); significant improvement in ACR50 and ACR70; Inflammatory response: decrease in CRP, ESR;	Injection site reactions (65% vs. 24% placebo) The rate of severe infections is similar to placebo.	Phase III	ISRCTN15819795	Cohen et al. (2004)
		Safety analysis was the main focus, no specific efficacy indicators were clearly defined.	Injection site reactions (72.6% vs. 32.9%) Severe infection (2.1% vs. 0.4%).	Phase III		Tesser et al. (2004)
		Efficacy Indicators: ACR50 response rate (31% vs. 41%);	Severe infection (0% vs. 3.7%–7.4%). Injection site reaction. Neutropenia.	Phase II	The European Group of Clinical Investigators	Genovese et al. (2004)
		Efficacy indicators: total Sharp score significantly decreased (at 48 weeks). Inflammatory response: CRP decreased. Efficacy indicators: maintain disease activity. Joint erosion: slowing progression.	Not Applicable.	Phase III	The Anakinra 960180 Study Group	Bresnihan et al. (2004)
		Not applicable.	Severe infection. Injection site reactions. Malignant tumors.	Phase III	The 990757 Study Group	Fleischmann et al. (2006)
		Efficacy indicators: ACR20 (64%), ACR50 (38%), ACR70 (17%). Inflammatory response: CRP, ESR improved.	Secondary failure (21.4%) Other adverse reactions were not detailed.	Phase II	The 990145 Study Group	Bao et al. (2011)
		Efficacy indicators: ACR20, laboratory parameters improved. Inflammatory response: IL-17, IFN-γ, IL-1β decreased.	Not Applicable.	Phase II	The 990757 Study Group	Niu et al. (2011)
Rilonacept	IL-1β soluble receptor	Efficacy indicators: ACR30 response rate higher (57% in rilonocept group vs. 27% in placebo group). Inflammatory response: shortened fever relief time.	Elevated liver enzymes (common), sJIA deterioration (4 severe events).	Phase II	The 20000223 Study Group	Ilowite et al. (2014)
Canakinumab	High-affinity IgG1 monoclonal antibody against IL-1β inhibits IL-1β binding to IL-1R1.	Efficacy indicators: ACR50 response rate significantly improved (26.5% vs. 11.4%). Inflammatory response: DAS28, HAQ score improved.	Low infection risk, rare injection site reactions.	Phase II	The European Group of Clinical Investigators	Alten et al. (2011)
Tadekinig alfa (IL-18BP)	IL-18 binding agents	Efficacy indicators: 50% of patients experienced a ≥50% decrease in CRP and fever subsided.	Mild injection reactions. 1 severe event (toxic optic neuropathy).	Phase II		Gabay et al. (2018)

**TABLE 3:** Targeting IL-1 family cytokines with monoclonal antibodies for the treatment of autoimmune diseases in clinical trials.

Target	Medicine	Mechanism	Disease	Clinical trial	Effect	NCT	References
IL-1α	Bermekimab	The monoclonal antibody	Hidradenitis suppurativa	II	The drug could achieve a HiSCR of 61%, and could effectively reduce inflammatory response (60%, 46%) and pain (64%, 54%) in patients with and without anti-TNF treatment, respectively.	NCT03512275	Gottlieb et al. (2020)
	Rilonacept	The IL-1 soluble receptor	Recurrent Pericarditis	III	This can rapidly relieve recurrent pericarditis attacks and significantly reduce the risk of recurrent peric arditis.	NCT03737110	Klein et al. (2021)
IL-1β	Canakinumab	The monoclonal antibody	Chronic Spontaneous Urticaria	II	Canakinumab lacks efficacy in the treatment of moderate to severe chronic spontaneous urticaria in adults.	NCT01635127	Maul et al. (2021)
IL-1Ra	Anakinra	An IL-1Ra analog,the IL-1 receptor antagonist	Gout Flares	II	It effectively relieves pain and has a good safety profile.	NCT03002974	Saag et al. (2021)
IL-18	Tadekinig alfa	IL-18BP	AOSD	II	Tadekinig alfa has a good safety profile and is beneficial for the early treatment of arthritis in patients with AOSD.	NCT02398435	Gabay et al. (2018)
IL-33	Astegolimab	A human IgG2 monoclonal antibody that selectively inhibits the IL-33 receptor ST2.	Severe asthma	IIb	It effectively lowers the annualized asthma exacerbation rate (AER) in patient populations, including those with low eosinophil levels and poor asthma control, with a good safety and tolerability profile.	NCT02918019	Kelsen et al. (2021)
	CNTO 7160	A monoclonal antibody against the IL-33 receptor	Asthma or atopic dermatitis	I	The drug has a safety and dose-dependent profile, but has no therapeutic effect in mild asthma and specific eczema.	NCT02345928	Nnane et al. (2020)
	Itepekimab	A monoclonal antibody targeting IL-33.	Asthma	II	Compared to placebo, itepekimab effectively reduced the occurrence of loss of asthma control, lowered eosinophil counts in the blood, and improved lung function in patients with moderate to severe asthma.	NCT03387852	Wechsler et al. (2021)
	Tozorakimab	A high-affinity humanized immunoglobulin G1 monoclonal antibody that can effectively neutralize IL-33.	COPD	I	The study was terminated due to lack of efficacy based on an interim analysis.	NCT03096795	Reid et al. (2024)
IL-36	Spesolimab	An anti-IL-36 receptor antibody.	Generalized Pustular Psoriasis	II	The drug has a rapid onset of action within 24 h and sustained efficacy for 12 weeks, with a safety profile similar to placebo. Some patients may require dose increases for treatment, but there are currently no clear identifiable markers.	NCT03886246	Elewski et al. (2023)
	Imsidolimab	A high-affinity humanized IgG4 monoclonal antibody (mAb) that can specifically bind to IL-36R and antagonize IL-36 signaling.	Generalized Pustular Psoriasis	II	The drug is efficacious with rapid and sustained effects, and has good tolerability and safety.	NCT03619902	Warren et al. (2023)

### 5.1 IL-1

Early administration of specific anti-IL-1β antibodies blocks IL-1β, thus inhibiting the expression of RANKL on regulatory T cells and suppressing osteoclast differentiation. This improves joint swelling and bone erosion in arthritis (Levescot et al., 2021). siRNA that binds to IL-1βsilences pro-inflammatory genes, which alleviate ankle joint swelling, bone erosion and cartilage destruction (Song et al., 2019).

hIL-1RA-Fc is an IL-1 receptor antagonist (IL-1Ra) analog that inhibits Th17 cell differentiation through blocking the STAT3 signaling pathway and induces Treg cell differentiation through the STAT5 signaling pathway, exerting anti-rheumatic arthritis effects. hIL-1RA-Fc can reduce the expression of IL-17, TNF-α, RANKL and VEGF, increase forkhead box P3 (Foxp3) gene expression, and thus suppress osteoclast genesis and angiogenesis (Lee S. Y. et al., 2016).

In a collagen-induced RA model, the soluble IL-1 receptor 2 receptor blocks IL-1α and IL-1β and significantly inhibits IL-1 signaling transduction in macrophages (Shimizu et al., 2015). Moreover, IL-1R2 inhibits Th17 cell activation by blocking IL-1β signaling transduction (Kim et al., 2021).

IgG26AW has high affinity, high neutralization ability and occupies a new binding epitope that binds both to IL-1RI and IL-1RAcP, which has been validated in tumors and may be a potential drug for RA in the future (Kuo et al., 2021).

### 5.2 IL-18

Currently, in research treating RA by targeting IL-18, inhibitors of IL-18 such as IL-18 binding protein (IL-18BP) are a major focus. IL-18BP can correct the imbalance of Th17/Treg cells in peripheral blood mononuclear cells from RA patients and reduce osteoclast genesis induced by IL-17 (Min et al., 2021). Consequently, IL-18BP promotes apoptosis of fibroblast-like synoviocytes while reducing apoptosis of chondrocytes, which is beneficial for the treatment of RA (Min et al., 2023).

Soluble IL-18 receptor beta (sIL-18Rβ) modulates Treg cells and Th17 cells similarly to IL-18BP, which suppresses collagen-induced arthritis (Veenbergen et al., 2010). This potential reduction in IL-18 expression has been associated with alleviation of RA symptoms in experimental models, though further investigation is needed to fully establish this pathway (Guo et al., 2022).

### 5.3 IL-33

Treatment with IL-33 neutralizing antibodies decreases levels of IFN-γ, IL-6, IL-12, IL-33, and TNF-α. This decrease significantly reduces the severity of joint damage (Li et al., 2020). The IL-33 specific receptor ST2 is also a therapeutic target. Blocking the ST2 receptor while raising IL-37 levels cooperatively dampens inflammation. This cooperation involves lowering expression of IL-6, TNF-α, toll-like receptors, and MMPs. It also inhibits M1 phenotypic polarization. Use of ST2 inhibitors to oppose IL-33 suppresses the stimulation of synoviocytes (Rai et al., 2022). It also decreases mRNA expression of RANKL and IP-10. Concurrently, ST2 inhibition elevates proinflammatory factor and MMP levels. It further boosts NF-κB activity, thereby inhibiting bone resorption (Lee E. J. et al., 2016).

### 5.4 IL-36 and IL-38

In an STIA mouse model, injection of IL-38 encoding was able to reduce the production of proinflammatory factors (including IL-17, IL-23, IL-22, TNF-α) by macrophages and synovial fibroblasts, lowering the inflammatory response in arthritic mice (Boutet et al., 2017). In rat models, overexpression of IL-38 and injection of IL-36 was able to target autophagy, regulating the proliferation, migration and invasion of synovial cells in RA (Hao and Liu, 2021).

However, some studies have also found that prophylactic treatment of mice with an IL-36R blocking antibody did not change clinical onset or disease patterns. Additionally, blocking IL-36 signal transduction did not alter the histopathological features of arthritis induced by TNF (Derer et al., 2014).

### 5.5 IL-37

IL-37 has intrinsic anti-inflammatory effects. IL-37 alleviates RA by inhibiting the production of IL-17 and IL-17-induced cytokines, and restricting the proliferation of Th17 cells (Ye et al., 2015). In an arthritis mouse model, low doses of recombinant IL-37 were able to inhibit 51.7% of arthritic inflammation, promote IL-1R expression, and reduce the synovial levels of IL-1β, IL-6, TNF-α, CXCL1, CXCR3, macrophage inflammatory protein 1-alpha (MIP-1α), IL-1α, and myeloperoxidase (MPO), thereby decreasing the recruitment of neutrophils to the joints (Cavalli et al., 2016).

### 5.6 Cytokine redundancy and therapeutic strategies targeting IL-1RAcP

Cytokine redundancy refers to the phenomenon where distinct cytokines bind to identical or structurally similar receptors, activating overlapping signaling pathways to elicit analogous biological functions. This redundancy enhances the adaptability and robustness of the immune system during pathogen invasion or tissue damage, ensuring core immune responses are maintained even if one cytokine pathway is blocked. Within the IL-1 family, proinflammatory cytokines (e.g., IL-1α, IL-1β, IL-18, IL-33, IL-36α/β/γ) rely on IL-1RAcP as a shared co-receptor to form a signal-transducing complex, thereby mediating common downstream pathways.

Studies in atherosclerosis highlight the functional overlap between IL-1α and IL-1β, which jointly regulate extracellular matrix-remodeling enzymes. Single-target inhibition of either cytokine has shown limited efficacy in this context (Beltrami-Moreira et al., 2016). Similarly, cytokine redundancy complicates therapeutic outcomes in sepsis, where IL-1 and IL-6 both activate STAT3, and TNF-α synergizes with IL-1 via the NF-κB pathway (Cooney and Yumet, 2002). These observations suggest that monotherapies targeting individual cytokines may yield suboptimal results in diseases like RA, necessitating strategies to disrupt shared receptor signaling.

**FIGURE 2:**
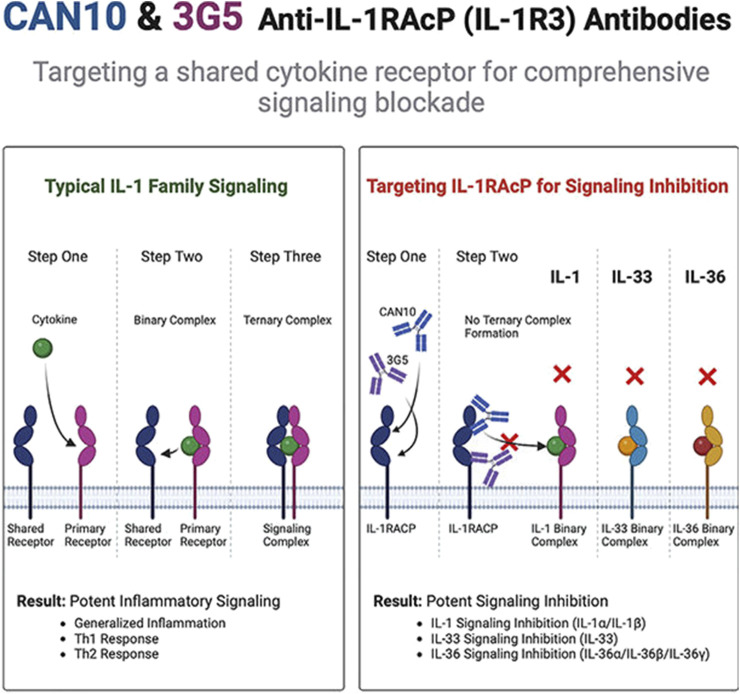
CAN10 and 3G5, two anti-IL-1RAcP antibodies, target distinct epitopes on this shared receptor and potently block IL-1α, IL-1β, IL-33, IL-36α, IL-36β, and IL-36γ signaling (Fields et al., 2024).

Addressing this challenge, Fields et al. demonstrated the feasibility of targeting IL-1RAcP to broadly block signaling by all IL-1 family cytokines (Figure 2) (Fields et al., 2024). Antibodies CAN10 and 3G5 specifically bind distinct epitopes (the c2d2 loop of the D2 domain and the D3 domain) on IL-1RAcP, effectively inhibiting cytokines dependent on this co-receptor, including IL-1α, IL-1β, IL-33, and IL-36α/β/γ. In acute peritonitis models, IL-1RAcP-targeted antibodies markedly reduced inflammatory cell infiltration and proinflammatory mediators (e.g., IL-6, G-CSF) compared to IL-1Ra, a natural antagonist of IL-1 signaling. Notably, CAN10 exhibited >10-fold higher inhibitory potency than IL-1Ra in suppressing IL-1α/β pathways. These findings underscore the therapeutic potential of targeting IL-1RAcP for IL-1-driven inflammatory disorders, particularly RA.

## 6 Clinical efficacy and adverse effects of IL-1 inhibitors

### 6.1 Current clinical trial results of IL-1 inhibitors

**FIGURE 3:**
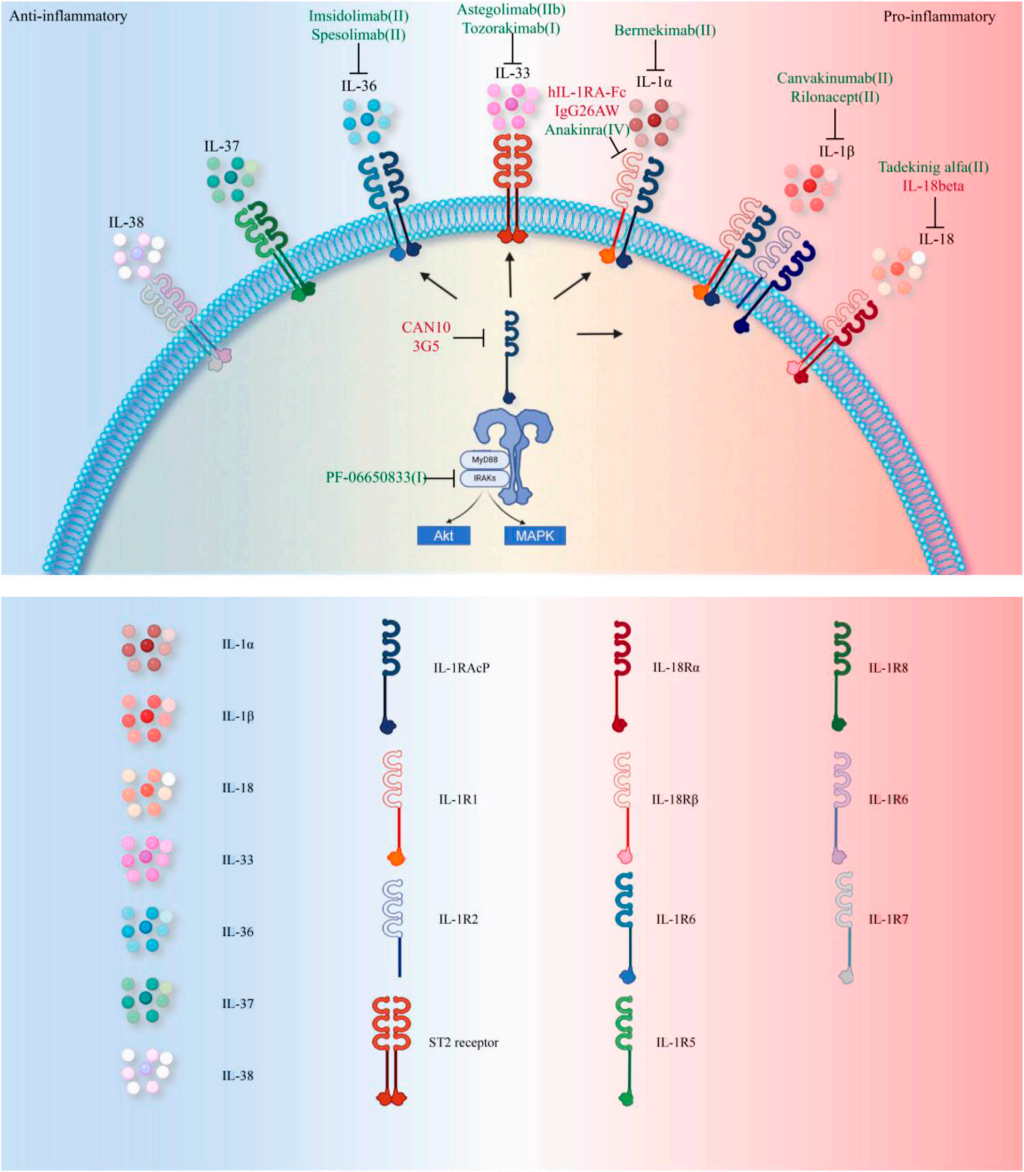
Therapeutic Strategies Targeting the IL-1 Family in RA. Currently, FDA-approved therapies primarily target the IL-1 pathway (annotated in green in the figure, with specific reference to RA clinical trial phases). Approved agents include: Anakinra (IL-1R1 antagonist), Canakinumab (IL-1β specific monoclonal antibody), Rilonacept (IL-1β trapping fusion protein), and Spesolimab (IL-36 receptor targeting monoclonal antibody approved for psoriasis). Investigational drugs in clinical trials address broader inflammatory targets: Bermekimab (IL-1α neutralization), PF-06650833 (IRAK4 inhibitor blocking downstream signaling), Tadekinig alfa (Th17 balance modulation via IL-18BP), Astegolimab and Tozorakimab (targeting IL-33 receptor ST2 and IL-33, respectively), Imsidolimab (IL-36R antagonism, analogous to Spesolimab). Preclinical studies (highlighted in red in the figure) explore novel mechanisms: hIL-1RA-Fc fusion protein enhances Treg/Th17 balance; CAN10 and 3G5 inhibit IL-1RAcP, a shared receptor subunit for IL-1 family cytokines; IL-38 gene therapy suppresses inflammation by inhibiting the AP-1/SIRT1 pathway; Recombinant IL-37 activates the Nrf2-mediated anti-inflammatory cascade; IgG26AW blocks IL-1α activity.

As previously established, IL-1 family cytokines play a central role in inflammatory responses and joint destruction in RA by activating synovial cells, promoting osteoclast activity, and inhibiting cartilage repair. Based on these mechanisms, multiple IL-1 inhibitors have been developed and tested in clinical trials. Table 1 summarizes the current biologics targeting IL-1 family cytokines. Figure 3 illustrates the therapeutic strategies for RA treatment using these agents.

To date, the IL-1 inhibitors approved for RA treatment include PF-06650833, Anakinra, Rilonacept, and Canakinumab. Among these, Anakinra—a recombinant IL-1 receptor antagonist—remains the first and only IL-1 inhibitor specifically approved for RA. Clinical trials since 1998 have yielded mixed results regarding its efficacy and safety. For instance, Scott et al. reported that short-term Anakinra therapy (12 months) only improved ACR20 and ACR50 response rates compared to placebo, with no significant differences in other efficacy endpoints (Scott et al., 2016). However, multiple studies demonstrated that Anakinra reduces DAS28 scores and lowers CRP/ESR levels (Nordström et al., 2012; Ruscitti et al., 2019; Ruscitti et al., 2021). Long-term use of Anakinra also slows joint erosion, as evidenced by decreased Sharp scores, though it shows no significant improvement in Larsen scores (Bresnihan et al., 2004). These findings collectively suggest that Anakinra exhibits moderate anti-inflammatory effects with long-term joint protection, making it a viable option for patients with inadequate responses to TNF-α inhibitors or those requiring infection-safe therapies (Scott et al., 2016).

The primary limitation of Anakinra is its high injection frequency (100 mg/day), leading to frequent injection-site reactions and poor patient compliance. Nevertheless, its infection risk remains low, with no reports of severe opportunistic infections, offering a safety advantage over TNF-α inhibitors (Fleischmann et al., 2006). Future directions include developing sustained-release formulations to reduce dosing frequency and identifying biomarkers (e.g., IL-1β levels or genetic polymorphisms) to predict treatment responses and optimize patient selection (Akash et al., 2013; Hong et al., 2024). Prolonged IL-1 blockade disrupts innate immune defenses, increasing bacterial infection susceptibility through impaired neutrophil homeostasis. Clinical surveillance data from long-term anakinra therapy demonstrate cancer incidence rates comparable to age-matched general population estimates, with no causative association identified between anakinra exposure and malignancy development (Fleischmann et al., 2006).

Canakinumab, a monoclonal antibody targeting IL-1β, has shown superior efficacy in early trials, with an ACR50 response rate of 26.5% and rapid improvements in DAS28 and HAQ scores (Alten et al., 2011). Its dosing regimen (every 4–8 weeks) and low incidence of injection-site reactions make it suitable for long-term maintenance therapy. However, clinical data on Canakinumab in RA remain limited, warranting further investigation. Other agents like Rilonacept and Tadekinig alfa lack robust RA-specific data but show potential in systemic inflammatory diseases and animal models, necessitating additional human trials.

Long-term administration of IL-1 inhibitors in RA clinical trials primarily manifests as injection site reactions and elevated infection risks. Emerging evidence from other immune-mediated conditions suggests additional class-related adverse effects that warrant consideration in RA treatment paradigms. Hepatotoxicity represents a notable concern, with 3%–8% of anakinra recipients developing transient liver enzyme elevations that typically resolve spontaneously (Ruperto et al., 2012). The lower incidence of hepatic dysfunction observed with canakinumab and rilonacept may reflect reduced metabolic stress from their prolonged half-lives (Grevich and Shenoi, 2017). Hematologic monitoring proves crucial as 2%–5% of anakinra-treated patients develop reversible leukopenia, potentially mediated by bone marrow suppression mechanisms (Minoia et al., 2018). While rare, macrophage activation syndrome (MAS) requires particular vigilance given its association with all three IL-1 blockers (Ravelli et al., 2016). This life-threatening complication, though inherent to AOSD pathophysiology, may be precipitated by therapeutic immune modulation (Junge et al., 2017). Serious infections including sepsis and pneumonia necessitate immediate drug cessation and antimicrobial therapy (Shimizu et al., 2013). Immunogenicity profiles vary significantly among agents. Anakinra’s recombinant structure predisposes to neutralizing antibody formation with consequent efficacy loss (Wikén et al., 2018), whereas the fully human canakinumab demonstrates no such immunogenic propensity (Pascual et al., 2005). Current literature remains inconclusive regarding long-term immunosuppression risks such as opportunistic infections or malignancy development. Nevertheless, cumulative biological effects mandate sustained surveillance to ensure therapeutic safety.

### 6.2 Comparative analysis of IL-1 inhibitors with other biologics

**FIGURE 4:**
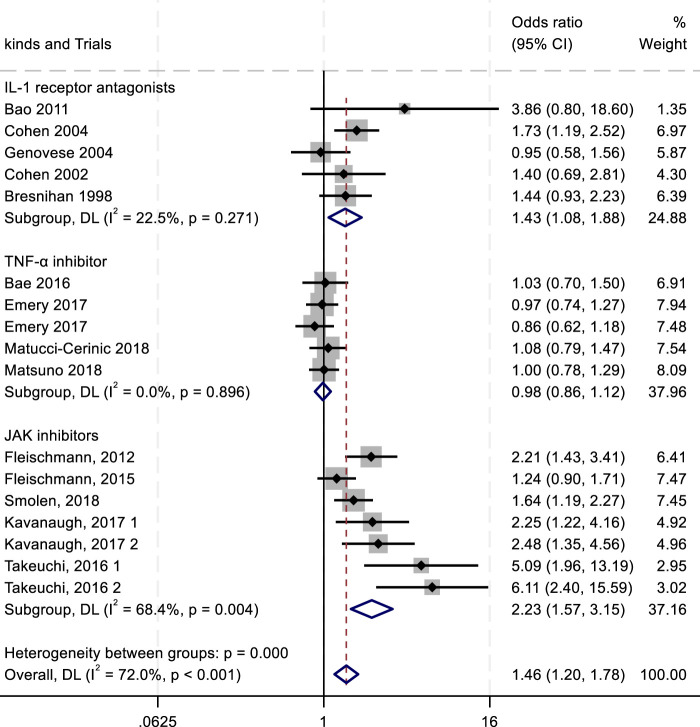
Results of a meta-analysis of ACR20 after 24 weeks of drug therapy.

**FIGURE 5:**
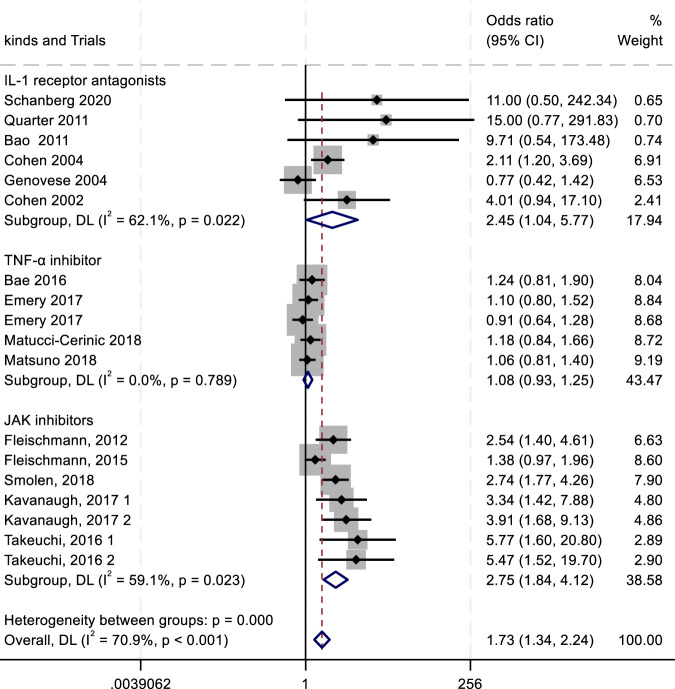
Results of a meta-analysis of ACR50 after 24 weeks of drug therapy.

**FIGURE 6:**
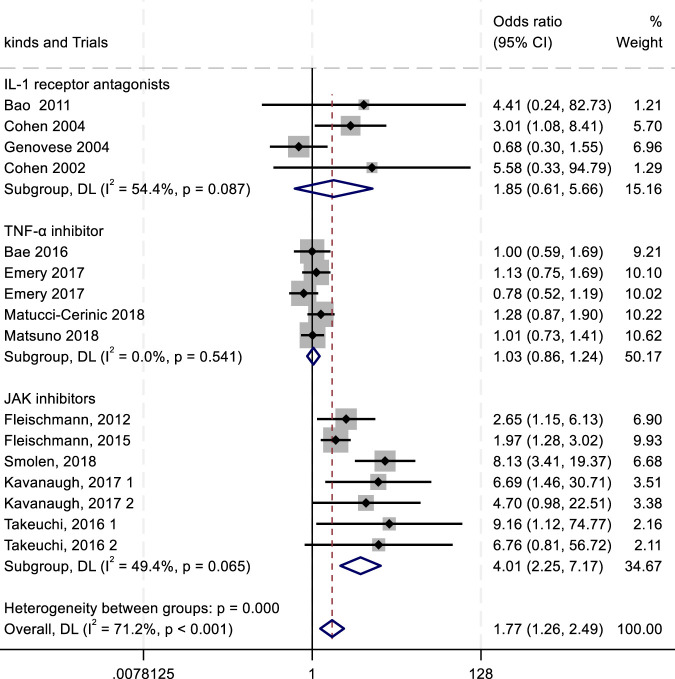
Results of a meta-analysis of ACR70 after 24 weeks of drug therapy.

**FIGURE 7:**
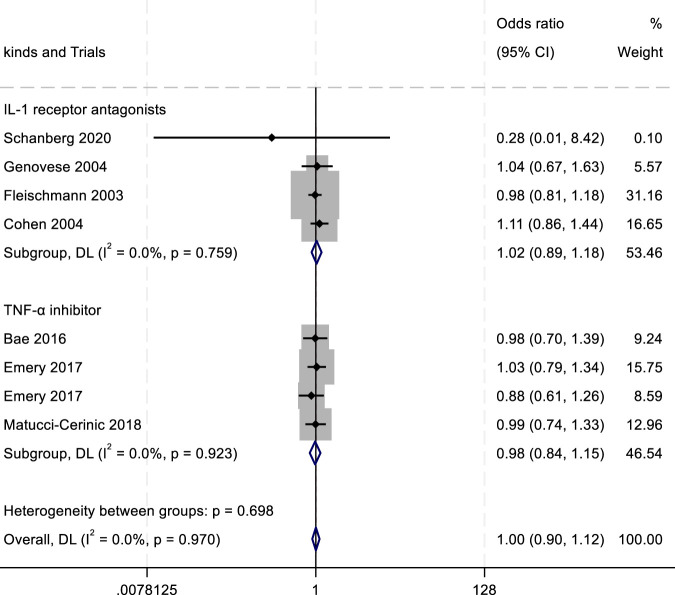
Results of a meta-analysis of the outcomes of adverse drug reactions after drug therapy.

**FIGURE 8:**
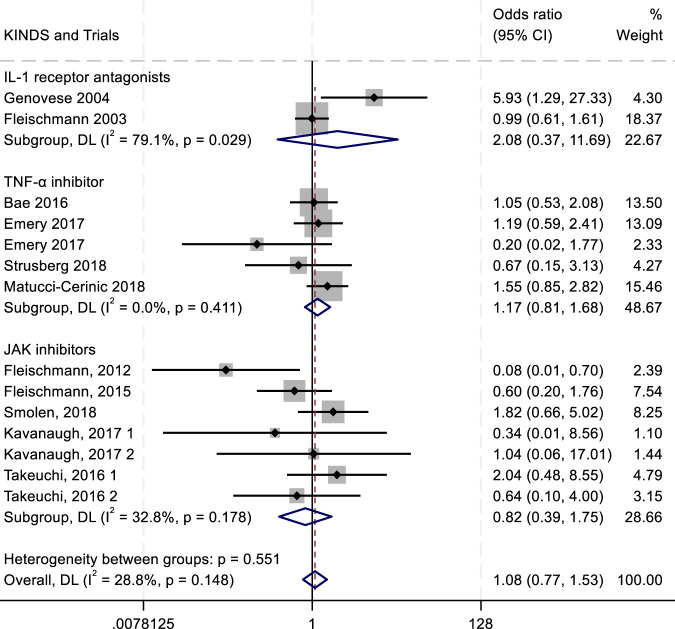
Results of a meta-analysis of the outcomes of serious adverse drug reactions after drug therapy.

To evaluate the efficacy and safety of IL-1 receptor antagonists (e.g., Anakinra) (Vasconcelos et al., 2020; Kazmi et al., 2024), TNF-α inhibitors (Hu et al., 2023), and JAK inhibitors (Ho Lee and Gyu Song, 2020), we conducted a meta-analysis comparing outcomes such as ACR20/50/70 response rates and adverse drug reactions (ADRs, serious ADRs, and treatment discontinuation due to adverse events). Key findings are summarized in Figures 4–8.

The ACR criteria response rates were utilized for efficacy evaluation. Overall, IL-1 inhibitors, TNF-α inhibitors, and JAK inhibitors demonstrated therapeutic benefits in reducing RA activity, showing associations with higher ACR20, ACR50, and ACR70 response rates. Among all treatment groups, JAK inhibitors exhibited the highest ACR20 response rate (RR 2.23; 95% CI 1.57–3.15; *P* < 0.004), with moderate heterogeneity (*I* ^2^ = 68.4%; *P* = 0.004). IL-1 inhibitors followed, showing an ACR20 response rate (RR 1.43; 95% CI 1.08–1.88; *P* = 0.271) with low heterogeneity (*I* ^2^ = 22.5%; *P* = 0.271). TNF-α inhibitors displayed the lowest ACR20 response rate (RR 0.98; 95% CI 0.86–1.12; *P* = 0.896) and minimal heterogeneity (*I* ^2^ = 0.00%; *P* = 0.896) (Figure 4). In the analysis of ACR50 response rates, the RR values were 2.75 for IL-1 inhibitors, 2.45 for TNF-α inhibitors, and 1.08 for JAK inhibitors (Figure 5). Similarly, for ACR70 response rates, the RR values were 1.85 for IL-1 inhibitors, 1.03 for TNF-α inhibitors, and 4.01 for JAK inhibitors (Figure 6). These patterns aligned with the ACR20 response rate trends. The findings suggest that JAK inhibitors demonstrate superior therapeutic efficacy, followed by IL-1 inhibitors, with TNF-α inhibitors showing the least effectiveness.

**FIGURE 9:**
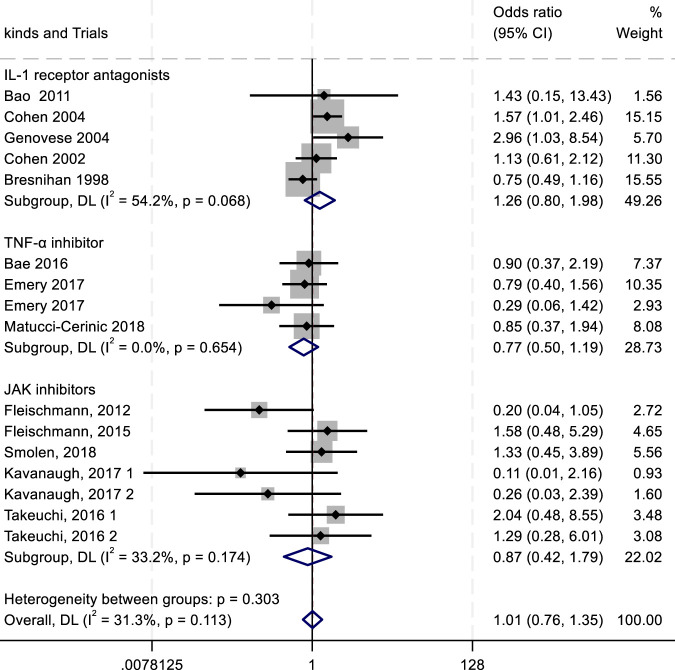
Results of a meta-analysis of withdrawal after drug therapy.

In the assessment of adverse events, the outcomes of adverse drug reactions (ADRs), serious adverse drug reactions (SADRs), and withdrawal due to adverse events were analyzed. For ADRs, the highest incidence was observed with IL-1 inhibitors (RR = 1.02; 95% confidence interval CI, 0.89–1.18; *P* = 0.759; *I* ^2^ = 0.00%), followed by TNF-α antagonists (RR = 0.98; 95% CI, 0.84–1.15; *P* = 0.923; *I* ^2^ = 0.00%) (Figure 7). Both drug classes showed numerically higher ADR rates compared with the placebo group. For SADRs, IL-1 inhibitors demonstrated the highest incidence (RR = 2.08; 95% CI, 0.37–11.69; *P* = 0.029; *I* ^2^ = 79.1%), with substantial heterogeneity across studies. In contrast, TNF-α inhibitors (RR = 1.17; 95% CI, 0.81–1.68; *P* = 0.411; *I* ^2^ = 0.00%) and JAK inhibitors (RR = 0.82; 95% CI, 0.39–1.75; *P* = 0.178; *I* ^2^ = 32.8%) exhibited lower SADR rates (Figure 8). The withdrawal outcomes followed a similar trend. IL-1 inhibitors again showed the highest withdrawal rates (RR = 1.26; 95% CI, 0.80–1.98; *P* = 0.068; *I* ^2^ = 54.2%), while TNF-α antagonists (RR = 0.77; 95% CI, 0.50–1.19; *P* = 0.654; *I* ^2^ = 0.00%) and JAK inhibitors (RR = 0.87; 95% CI, 0.42–1.79; *P* = 0.174; *I* ^2^ = 33.2%) had lower withdrawal rates (Figure 9). In summary, IL-1 inhibitors were associated with a higher incidence of adverse drug reactions compared with JAK inhibitors and TNF-α antagonists.

IL-1 inhibitors exhibit unique advantages in RA subtypes with autoinflammatory features. Their superior ACR50/70 response rates compared to TNF-α inhibitors may stem from selective blockade of innate immune pathways (e.g., IL-1β), particularly benefiting subgroups with periodic fever or NLRP3 mutations. However, their overall efficacy lags behind JAK inhibitors (notably in ACR20/70 responses), and safety concerns—including higher rates of severe adverse events and treatment discontinuation—may relate to suppression of IL-1β’s broad physiological roles (e.g., infection defense). Future strategies should focus on biomarker-guided patient selection, optimized combination therapies, and targeted drug design. Integration of real-world data and AI-driven models will advance personalized treatment paradigms, enabling high-efficacy, low-toxicity outcomes in specific RA populations.

### 6.3 Therapeutic potential of IL-1 inhibitors

While IL-1-targeted therapies have limitations, they remain a critical component of RA treatment. For some patients, IL-1 inhibitors are used as adjunctive therapy after inadequate responses to methotrexate (MTX) or TNF-α inhibitors, or as part of combination regimens. However, IL-1 inhibitors may be prioritized as first-line therapy in the following patient subgroups:

#### 6.3.1 Early intervention treatment for RA

Current evidence demonstrates superior clinical efficacy of IL-1 inhibitors when administered during early-stage RA. In treatment-naïve patients with active disease, IL-1 receptor antagonists like anakinra achieve substantial symptom improvement, with 43% of recipients attaining ACR20 response criteria and 44% meeting Paulus remission standards within initial therapeutic windows. Clinical trials document significant reductions in joint swelling counts, tender joint indices, and CRP levels following early intervention (Bresnihan et al., 1998). Combination therapy with methotrexate enhances treatment response, yielding 38% ACR20 achievement versus 22% in placebo controls (p < 0.001) after 24 weeks (Cohen et al., 2004). Radiographic assessments reveal dose-dependent protection against structural damage, with 150 mg/day anakinra regimens demonstrating significantly reduced modified Sharp scores compared to placebo (p = 0.015) (Bresnihan et al., 2004). Advanced imaging analyses confirm therapeutic advantages through diminished Larsen scores and fewer eroded joints in early-treatment cohorts, supporting the critical window for IL-1 blockade in disease modification (Bresnihan et al., 1998).

The therapeutic landscape shifts dramatically in established RA, where irreversible joint damage and complex inflammatory networks limit IL-1 inhibitor efficacy. Radiographic studies show persistent elevation of modified Sharp scores (p = 0.015) despite 48-week anakinra treatment in patients with baseline structural damage, indicating irreversible osteochondral destruction (Bresnihan et al., 2004). The cytokine redundancy characteristic of late-stage disease undermines monotherapy effectiveness, as evidenced by equivalent ACR50 response rates between anakinra/etanercept combination therapy (31%) and TNF inhibitor monotherapy (41%) in methotrexate-refractory cases (Genovese et al., 2004). This therapeutic plateau reflects TNF-α and IL-6 pathway dominance that bypasses IL-1 blockade mechanisms. Combination strategies introduce heightened safety concerns, with severe infection rates escalating to 3.7%–7.4% in dual biologic regimens compared to null events in monotherapy controls (Genovese et al., 2004). Longitudinal data reveal persistent disease progression (67.80 events/100 patient-years) despite sustained IL-1 inhibition, suggesting cumulative immune cell exhaustion mechanisms (Fleischmann et al., 2006). The compromised bone marrow reserve in advanced RA may further diminish therapeutic response through altered leukocyte dynamics.

#### 6.3.2 RA subtypes with high IL-1β expression or IL-1RN2 allele mutations

Yuan et al. employed Mendelian randomization to analyze the role of IL-1 signaling in RA. They found that seropositive RA was more closely associated with IL-1β, IL-1 receptor antagonist (IL-1Ra), and IL-6, whereas seronegative RA correlated with IL-2ra, IL-8, and IL-18 (Yuan et al., 2022). The association between IL-1β and seropositive RA was primarily driven by single nucleotide polymorphisms (SNPs) in the HLA-DQA1 region. Sensitivity analyses confirmed robust results with no horizontal pleiotropy, supporting the direct pro-inflammatory role of IL-1β and the protective antagonism of IL-1Ra. These findings suggest that sustained IL-1 inhibition or enhanced IL-1ra activity may reduce RA risk, particularly in seropositive subtypes. Reverse MR analysis further indicated that RA may promote downstream IL-6 signaling, providing a rationale for combined targeting of IL-1 and IL-6.

#### 6.3.3 RA patients with autoinflammatory syndromes

IL-1 inhibitors demonstrate favorable safety and efficacy in managing autoinflammatory conditions such as Kawasaki disease, idiopathic recurrent pericarditis, Behçet’s disease, monogenic autoinflammatory diseases (AIDs), undifferentiated AIDs, chronic non-bacterial osteomyelitis, macrophage activation syndrome, and febrile infection-related epilepsy (Maniscalco et al., 2020; Del Giudice et al., 2022; Alexeeva et al., 2023). For severe or recurrent cases, IL-1 inhibitors should be considered a valuable therapeutic option in pediatric populations with rare inflammatory disorders.

#### 6.3.4 Patients with metabolic comorbidities

Clinical studies comparing Anakinra and TNF-α inhibitors (TNFi) revealed distinct benefits in RA patients with type 2 diabetes. Anakinra significantly improved metabolic parameters (e.g., HbA1c) and inflammatory markers (e.g., DAS28), with sustained effects during long-term follow-up. These improvements reduced the need for antidiabetic medications, whereas TNFi showed no such metabolic benefits (Ruscitti et al., 2021). This positions Anakinra as a superior choice for RA patients with concurrent metabolic disorders.

#### 6.3.5 Patients at high cardiovascular risk

Systemic inflammation in RA contributes to endothelial dysfunction and atherosclerosis, underscoring the importance of inflammation control for reducing cardiovascular risk (Weber et al., 2023). IL-1 inhibitors have demonstrated efficacy in lowering inflammation and improving cardiovascular outcomes, making them a promising option for high-risk RA patients (Dragoljevic et al., 2020). Although JAK inhibitors are effective in RA, their association with elevated venous thromboembolism risk limits their use in this population (Goldman et al., 2024). While TNF inhibitors reduce cardiovascular events in RA, their efficacy varies, and some patients exhibit inadequate responses (Desai et al., 2014; Sattin and Towheed, 2016; Hsieh et al., 2020). IL-6 inhibitors also show potential in cardiovascular risk reduction, though long-term data remain limited (Gerasimova et al., 2024).

## 7 Discussion

Existing drugs for the treatment of RA face challenges related to drug tolerance, incomplete efficacy, and patient heterogeneity. As a result, research has increasingly focused on cytokine-targeted therapies that align with the immune mechanisms underlying RA (Cavalli and Dinarello, 2015). Among these, the IL-1 family plays a significant role in disease pathogenesis by engaging distinct receptors and activating TIR-mediated Akt and MAPK signaling pathways, ultimately influencing inflammatory factor expression (Dinarello, 2018). The complex interplay within this cytokine family includes both pro-inflammatory members—such as IL-1α, IL-1β, IL-18, IL-33, IL-36α, IL-36β, and IL-36γ—and anti-inflammatory members—including IL-1Ra, IL-36Ra, IL-37, and IL-38. This intricate balance suggests that targeted modulation of IL-1 family cytokines, using soluble cytokine receptors, cytokine antagonists, and cytokine analogues, holds promise for RA treatment.

However, current research targeting the IL-1 family is relatively limited. In clinical trials, multiple antibodies have not been studied, and the studies that have been conducted have not shown ideal therapeutic effects on RA, often being used for RA that is ineffective to other drug treatments or in combination with other drugs to treat RA. The reasons for the poor efficacy may include the following:

### 7.1 Cytokine redundancy and compensation mechanisms

One of the primary challenges in targeting a single cytokine within the IL-1 family is the presence of functional redundancy. Many IL-1 family cytokines have overlapping roles, and inhibiting one member can lead to compensatory upregulation of others, thereby sustaining inflammation (Voronov et al., 2013). For example, blockade of IL-1β may lead to increased activity of IL-α, potentially undermining the efficacy of IL-1β-targeted therapies. This compensatory response has been observed in other inflammatory conditions, such as atherosclerosis, where cytokine inhibitors inadvertently modulate multiple inflammatory pathways, sometimes reducing treatment effectiveness (Beltrami-Moreira et al., 2016).

### 7.2 Limited clinical success of IL-1 inhibitors

While IL-1 inhibition has demonstrated efficacy in conditions such as systemic juvenile idiopathic arthritis and autoinflammatory syndromes, its impact in RA has been less pronounced (Pardeo et al., 2021; Arnold et al., 2022). Clinical trials investigating IL-1-targeting agents—such as anakinra (IL-1 receptor antagonist) and canakinumab (anti-IL-1β monoclonal antibody)—have not shown superiority over existing RA treatments like TNF-α inhibitors or IL-6 blockade (Singh et al., 2016). Consequently, IL-1 inhibitors are often reserved for patients who do not respond to other therapies or are used in combination regimens. Furthermore, IL-1 is a key mediator of innate immunity, and blocking its activity may weaken the body’s defense against pathogens. For instance, IL-1β activates neutrophils and macrophages to clear pathogens, and inhibiting its function may increase the risk of bacterial or viral infections. Treatment with IL-1 inhibitors may induce serious infections such as pneumonia and tuberculosis, especially more pronounced in patients with immunosuppression (Selmi et al., 2015). This may be a reason limiting the clinical application of IL-1.

### 7.3 Anti-drug antibodies (ADA) and immunogenicity

The human immune system can generate ADA against therapeutic monoclonal antibodies, potentially leading to treatment failure or hypersensitivity reactions. For instance, monoclonal antibodies such as infliximab, adalimumab, anakinra, and tocilizumab have been associated with ADA formation, reducing their therapeutic efficacy. Similarly, heterologous monoclonal antibodies, such as the rabbit-derived TNF-α antibody SSS07, have been reported to trigger ADA responses, limiting their clinical utility (Wang and Jin, 2020; Iwaszko et al., 2021).

### 7.4 Inter-individual variability and disease heterogeneity

RA exhibits significant inter-individual variability, influenced by genetic background, environmental factors, and disease subtypes. This variability affects cytokine expression patterns, making a one-size-fits-all therapeutic approach challenging. For example, racial differences have been shown to influence the efficacy of certain biologics, such as golimumab in ulcerative colitis (Greywoode et al., 2023). Personalised medicine approaches—such as gene and protein-level analyses—may be necessary to optimise IL-1-targeted therapies in RA (Lim et al., 2022).

### 7.5 Therapeutic potential of combined IL-1 family antagonists with other cytokine inhibitors in RA

The primary challenge of IL-1 family inhibitors in RA treatment lies in the limited efficacy of single-target inhibition due to cytokine network redundancy and compensatory mechanisms. Combining these inhibitors with other cytokine blockers (e.g., TNF-α or IL-6 antagonists) or developing multi-target drugs may enhance therapeutic outcomes through the following mechanisms: 1) IL-1 family cytokines primarily activate the TIR-MyD88-Akt/MAPK pathway, driving inflammatory responses in innate immune cells. In contrast, TNF-α and IL-6 mediate adaptive immune changes and systemic inflammation via the NF-κB and JAK-STAT pathways, respectively. Combination therapy inhibits these pathways synergistically, reducing cross-induction of pro-inflammatory factors and improving disease control. Notably, IL-1 inhibitors and TNF-α/IL-6 antagonists share overlapping pathways; for example, combining IL-1 inhibitors with TNF-α inhibitorss can cooperatively suppress synovial fibroblast activation and bone erosion, potentially achieving deeper remission. 2) Single-agent IL-1β blockade may trigger compensatory increases in IL-18 or IL-36γ. However, combining IL-1 inhibitors with TNF-α blockers or IL-6 receptor antagonists can counteract these compensatory signals and prolong clinical remission. 3) Targeting two IL-1 family cytokines simultaneously neutralizes dual pro-inflammatory isoforms, minimizing therapeutic resistance caused by functional redundancy and enabling comprehensive suppression of inflammation. This integrated approach highlights the potential of IL-1-targeted combination therapies to address RA heterogeneity while emphasizing the need for rigorous safety evaluation to balance efficacy and infection risks.

## 8 Prospects

Targeting the IL-1 family has improved symptoms of RA, but some issues remain to be addressed. In the future, we can conduct in-depth research from the following perspectives: First, develop multi-specific antibodies that can target multiple IL-1 family cytokines simultaneously, to enhance efficacy. Second, use human monoclonal antibodies to replace existing murine antibodies in order to mitigate immune-related adverse reactions. Third, explore the combined application of IL-1 inhibitors with conventional therapies such as DMARDs, which may significantly enhance treatment effects and shorten treatment cycles. Last but not least, develop personalized treatment regimens tailored to different subtypes of patients based on their genetic profiles and protein expression characteristics, to achieve precision medicine. In the future, further research is believed to be able to improve the level of IL-1 targeted therapy for RA and bring about better clinical outcomes for patients.
